#  When the present can help refine interpretations of the past: covid-19 in real time and the historiography of epidemics 

**DOI:** 10.1590/S0104-59702023000100041

**Published:** 2023-09-04

**Authors:** Diego Armus

**Affiliations:** i Swarthmore College. Swarthmore – PA – USA darmus1@swarthmore.edu

**Keywords:** Covid-19, History, Epidemics, Historiography, Covid-19, Historia, Epidemias, Historiografía

## Abstract

These notes address the proliferation of discourses with improvised, uninformed, apocalyptic and voluntarist approaches. They emphasize issues such as the widespread ignorance about the history of epidemics, and the inability to deal with the uncertainties that reign during pandemic times, as well as the announcements that this extraordinary health/sanitary event would produce a profound watershed in all walks of life and in all corners of the world. Finally, these notes seek to point out how the present can illuminate the study of the past – or, more personally, what I think I have learned as a historian in the times of the covid-19 pandemic.

## Resumen

Estas notas abordan la proliferación de discursos con enfoques improvisados, desinformados, apocalípticos y voluntaristas. Enfatizan cuestiones como la ignorancia sobre la historia de las epidemias, y también la incapacidad para lidiar con las incertidumbres que reinan durante las pandemias, así como los anuncios que este extraordinario evento sanitario produciría un profundo parteaguas en todos los órdenes de la vida y en todos los rincones del mundo. Finalmente, estas notas buscan señalar cómo el presente puede iluminar el estudio del pasado – o, de modo más personal, lo que creo haber aprendido como historiador en los tiempos de la pandemia de la covid-19.

Con el telón de fondo de la pandemia de covid-19 me gustaría reflexionar sobre el trabajo de los historiadores frente al desafío de narrar la historia de las epidemias.

Cuando recién se había desatado la pandemia de covid-19, el entonces presidente estadounidense Donald Trump se preguntó: "¿Quién podría haberlo sabido?". La transparente brutalidad e ignorancia de Trump, lo sabemos, es de él. Pero la sorpresa, lamentablemente, ha sido y sigue siendo de muchos.

Sin embargo, ya a finales de la década del setenta epidemiólogos y virólogos comentaban con ironía que al siglo XIX lo siguió el siglo XX y al final del siglo XX y comienzos del XXI, lo sigue el siglo XIX. Era un modo de decir que las epidemias habían regresado de la mano de nuevos agentes patógenos o cepas renovadas de algunos ya conocidos.

En verdad, las epidemias nunca se habían ido del todo. Una desmedida confianza en la ciencia dominó el tercer cuarto del siglo XX, pero la llegada del sida, con todas las incertidumbres que trajo consigo, se ocupó de anunciar que serían décadas en que los brotes epidémicos no harían más que repetirse. Así llegaron las epidemias de Sars, H1N1, Mers y Ébola. A ellas se ha sumado la covid-19.

* * *

Las incertidumbres que marcan la experiencia individual y colectiva de vivir la pandemia alentaron una tremenda proliferación de reacciones y discursos. A todo lo largo del 2020 abundaron los vaticinios, algunos sinceros y legítimos, otros apocalípticos y esperanzados, muchos improvisados y apenas informados, casi todos convencidos que ya nada seguiría siendo tal como lo conocimos.

En esta proliferación de discursos hubo de todo: los filósofos, cientistas políticos e intelectuales públicos que hablaban sin dudar que se trataba de una "gripita" o que la cuarentena era una clara evidencia de la emergencia de Estados con vocación autoritaria (Agamben et al., 2020; Bagiotto Botton, 2020); los periodistas y opinadores que acababan de descubrir la epidemiología y pontificaban como expertos; los políticos obligados a reconocer la existencia de una crisis sanitaria que descubrían no solo las limitaciones que esa crisis imponía a la política sino también su incapacidad de sincerar frente a la sociedad las inevitables dudas y perplejidades que saturan los tiempos de epidemia, la cruel dinámica de la prueba y el error mientras se despliegan iniciativas para gobernar la pandemia. Algunos tópicos de esas intervenciones fueron memorables. Entre ellos los que lo explicaban todo por el neoliberalismo, desde una posible zoonosis, producto del salto de un virus de una especie a otra a la falta de agua potable para higienizarse. Otros articulaban explicaciones más culturalistas y anunciaban la emergencia de una muy disciplinada vida cotidiana, sin besos, porque las mascarillas habían llegado para quedarse. Y otros, con un notable voluntarismo, anunciaban la crisis terminal del capitalismo y apostaban a estar vislumbrando la aurora de una nueva y mucho más justa sociedad.

En 2021, y más aún en 2022, ganaron presencia las lecturas realistas y despojadas de ampulosidad. Se habla de los pocos recursos relativamente eficaces para mitigar el contagio, de las vacunas, del uso discrecional y limitado de la cuarentena y las medidas de distanciamiento social, de las mascarillas, de los testeos, de los tratamientos post-contagio.

* * *

A mucha gente – en puestos de gobierno y gestión, o dedicada a opinar e intervenir sobre las coyunturas del presente – la pandemia los invitó a bucear, a las apuradas, en la historia y los avatares de la salud pública, de la biomedicina y de la epidemiología retrospectiva. Por lo general sus disquisiciones comenzaban con el mantra de "aprender del pasado", de las epidemias del pasado en plural, ignorando la singularidad de cada epidemia.

En ese contexto, quienes nos dedicamos a la historia de la salud y la enfermedad fuimos interpelados con la pregunta de "qué dice la historia", una pregunta que parece sugerir que la historia funciona como una suerte de GPS frente al presente. Eso de la historia como escuela de la vida.

Soy de los que piensan que la historia no es escuela del presente, no da lecciones y no puede definir una detallada hoja de ruta para evitar equivocaciones. Solo puede ofrecer lineamientos generales, esbozar un sentido de complejidad de la experiencia social e individual en el pasado, apenas iluminar algunos asuntos que son difíciles de vislumbrar en la vorágine y urgencias del presente.

Entonces, más que la pregunta "se aprende del pasado" para entender el presente, me gustaría reflexionar sobre cómo el presente puede enseñarnos algo a los historiadores o, de modo más acotado y si se quiere personal, lo que yo aprendí en estos largos meses, aun inacabados, de pandemia.

Tal vez la primera lección puede condensarse en una línea: escribir y pensar una epidemia es mucho más fácil que vivirla. La pandemia ha sido y sigue siendo una maratón, plagada de incertidumbres y presumir que de la mano de las supuestas lecciones del pasado se puede navegar la neblina del presente es, me parece, un despropósito. Dicho de otro modo: tomar nota y hacerse cargo, explícitamente, de la infinidad de asuntos – muchos saturados de complejas ambigüedades – que no podemos ni siquiera problematizar en la historia de las epidemias. Y reconocer que esa lista es larga.

La tremenda complejidad y densidad de esta epidemia debe educarnos en la humildad. Tal vez podemos dejar claro que para algunos asuntos hemos encontrado algo así como una respuesta o articulado una interpretación más o menos convincente. Pero para muchos otros, solo nos quedan las preguntas. Parece una obviedad. Pero si recorremos la historiografía de las epidemias y las enfermedades no es difícil concluir en la necesidad de recordar este balance inevitablemente punteado por lagunas y simplificaciones. La metáfora que el historiador del arte Ernst Gombrich (27 dec. 1973) esbozó hace tiempo, eso de la historia como un queso suizo con agujeros, parece pertinente. Es mucho lo que no sabemos sobre las epidemias del pasado. Frente a esas lagunas podemos usar nuestra intuición y cierta sensibilidad, pero siempre constreñidos a la regla dura de la evidencia. Con esos presupuestos conjeturamos lo que pasó. Y como ocurre con muchos asuntos, las evidencias no siempre están disponibles. Entonces se trata de articular bien la pregunta, aun sin poder responderla. Volviendo a la metáfora de Gombrich: se trata de reconocer la existencia de los agujeros, porque son parte del queso.

La segunda lección resulta del riesgo de mirar el pasado como si fuera un anticipo del presente. La covid-19 nos ha revelado una batería de temas y cuestiones que saturan las multifacéticas respuestas – colectivas e individuales – desplegadas frente a las urgencias traídas por esta pandemia. Tenerlas presente es una cosa, pero trasladarlas mecánicamente, sin situarlas en un tiempo y espacio específicos, sin localizarlas, puede llevar a lecturas totalmente anacrónicas del pasado. Por ejemplo, enfatizar en todo lo que el Estado no ha hecho frente a una epidemia de fiebre amarilla a fines del siglo XIX cuando, en verdad, eran tiempos en que el Estado en los países latinoamericanos estaba recién desarrollando su agenda de salud pública o se sabía muy poco sobre esta enfermedad. O a la inversa, concluir en que la presencia de quince inspectores sanitarios es evidencia, en la década de 1940, de la medicalización y disciplinamiento de la vida y la muerte de los habitantes de una ciudad de un millón de personas, tal vez sean rasgos mucho más ostensibles de la experiencia urbana a fines del siglo XX. Son apenas dos ejemplos. Pero sobran lecturas anacrónicas, con frecuencia modeladas al calor de algún marco teórico de moda que sin duda puede ayudar a refinar la perspectiva, pero también puede terminar encorsetando y aplanando las especificidades temporales y geográficas.

La tercera lección subraya la necesidad de identificar lo que las epidemias tienen en común y lo que no tienen en común, las similitudes, las diferencias y las peculiaridades. Charles Rosenberg (1989) nos ha enseñado que hay una dramaturgia más o menos compartida por casi todas las epidemias y que sin duda hay fenómenos que parecen estar inevitablemente presentes en todas ellas. Pero la repetición, casi mecánica, de esa dramaturgia en la narrativa histórica de cualquier epidemia puede llevarnos muy fácilmente a ignorar la singularidad de cada una. Se trata entonces de localizar la epidemia, evitando no solo miradas trans-históricas, sino también las interpretaciones que usan la dimensión de "lo global" para explicar lo todo. En otras palabras: discutir las epidemias o pandemias, en plural y cada una en su especificidad.

La política durante la pandemia y las reacciones de la gente frente a la falta de respuestas eficaces de la medicina en materia de tratamientos son dos asuntos muy densos y complejos. Sobre esto, la cuarta lección: evitar explicaciones tremendamente simplistas respecto de la política en tiempos de epidemia. ¿Cómo reaccionan el Estado, la política y la sociedad? En la coyuntura epidémica, el Estado habla muchos libretos al mismo tiempo y esos libretos tienden a ser muy cambiantes porque las urgencias generadas por la epidemia cambian y no se mantienen estáticas. ¿Cuánta legitimidad logran los sistemas políticos en su intento de mitigar el contagio y controlar la epidemia? La coyuntura epidémica demanda consensos y convergencias entre los actores políticos: ¿se logran tales acuerdos básicos? ¿Esos consensos se materializan en intervenciones? En tiempos de profundas incertidumbres, las intervenciones demandan del Estado y de los gobiernos un muy sutil manejo de las expectativas colectivas, de la comunicación de las medidas que pueden – o no – mitigar el contagio, de la comunicación de los necesarios ajustes frente a equivocaciones que, por momentos, parecen inevitables. ¿Se trata de respuestas mínimamente convincentes y eficaces? Aquí vale la pena recordar que han sido muchas las epidemias en el pasado – tal vez la mayoría – que se apagaron solas, más allá de lo que el Estado, la salud pública, la medicina, la gente común y la ciencia pudieron haber hecho. ¿Y las reacciones de la sociedad frente a la incertidumbre y los fracasos? Allí está, es innecesario decirlo, el complejísimo mundo de las experiencias vividas, de los miedos al contagio y la muerte, de las reacciones individuales y colectivas, de la fatiga y el hartazgo frente a iniciativas de salud pública, difíciles de sostener por largos períodos o con resultados poco evidentes, de los tratamientos improvisados y de los tratamientos alternativos a la medicina oficial, de las experiencias previas forjadas en epidemias del pasado más o menos reciente. Y frente a esas reacciones y respuestas de la población, ¿con qué talante, con cuánta rigidez y rigor, con qué argumentos, se despliegan las respuestas del Estado? Por detrás de estas preguntas se prefiguran otras, muy relevantes: ¿Acaso la naturaleza de un cierto régimen político puede explicar las mejores o peores performances de la salud pública en la coyuntura epidémica? ¿O, más que el régimen político, se trata de la confianza de la sociedad en la capacidad del Estado de intervenir del mejor modo posible en los empeños por navegar la crisis sanitaria? ¿La solidez de las instituciones de salud pública es suficiente para confrontar la crisis sanitaria con relativo éxito o a ellas también hay que sumar otras instituciones y infraestructura que permiten efectivizar las iniciativas de salud pública? ¿Cómo se ajustan localmente consensos internacionales – en caso tales consensos existan – en un país o en sus diversas regiones, en particular cuando rige una estructura político institucional muy federal? ¿Cómo se ajustan localmente, tanto en las periferias como en los centros, las tensiones resultantes de la falta de consensos? ¿Cuánto cuentan las experiencias epidémicas anteriores, distintas, pero proveedoras de una suerte de entrenamiento institucional y logístico para lidiar con urgencias e incertidumbres?

La “quinta lección” se articula bien en una pregunta: ¿Cuándo termina una epidemia? La respuesta puede ser arbitraria, producto de una decisión política más o menos informada, producto del acostumbramiento a vivir con una nueva enfermedad que acarrea aceptables índices de morbilidad y mortalidad. A comienzos de la década de 1970, Michel Foucault (1977) publicó una extensa crítica a cualquier empresa que pretendiera alcanzar la verdad histórica descubriendo sus inicios elementales. Citando Nietzsche decía que la historia enseña a reírse de las solemnidades del origen enfatizaba que los orígenes históricos reales no fueron ni hermosos ni, en última instancia, muy significativos. Foucault señalaba el peligro de celebrar el evento inicial, olvidando el proceso. Y si esta advertencia parece pertinente para los momentos de iniciación, también lo es para los momentos de cierre, de finalización. Quiero decir, la estadística epidemiológica puede permitir aventurar el fin de un ciclo epidémico, pero hay mucho más que ese arbitrario umbral numérico.

El comienzo y final de una epidemia son asuntos saturados por complejas temporalidades. Enfermedad, endemia, epidemia, pandemia son etiquetas relativas y también son etiquetas políticas. La definición de pandemia o epidemia asociada a la irrupción sin aviso, por un tiempo corto y mucha muerte, es muy insatisfactoria. Y también lo son los momentos del comienzo y del cierre. Recordemos que estamos viviendo hace cuarenta años con la pandemia de HIV-sida. Recordemos que los ciclos de la tuberculosis fueron aún más largos y hubo tiempos en que se la etiquetó de epidemia y otros de endemia.

La sexta lección: las enfermedades y las epidemias son parte de la experiencia humana. Algunas terminarán controladas, deviniendo en enfermedades con las que se aprende a convivir. Otras desparecerán como resultado de exitosos esfuerzos de erradicación. Otras, como se dijo, se apagaron solas. Cada epidemia de enfermedades infectocontagiosas es única, resultante de un microorganismo y del modo en que una sociedad la confronta, reacciona e interpreta. Han sido, siempre, parte de la historia de la humanidad. Y lo seguirán siendo. Es cierto que algunas enfermedades son evitables y es particularmente irritante cuando no se hace lo que se debe para evitarlas. Pero no todas las enfermedades son evitables. Desde siempre las relaciones entre sociedad y medio ambiente han sido inestables. Muchas epidemias emergieron como resultado de un desequilibrio donde factores no humanos – como las mutaciones genéticas – o intervenciones humanas – como las alteraciones en el entorno medioambiental – se enhebraban con contextos sociales, políticos y culturales particulares. No me interesa subrayar ahora las necedades y horrores hechos por los hombres, sobre lo que hay abundante literatura. En cambio, quiero remarcar la existencia de procesos que no son resultado directo de las acciones del hombre. Y frente a los cuales la salud pública y la ciencia solo logran manejarse del mejor modo posible en la neblina de las incertidumbres. En el apéndice a la "Parte 1" de su *Ética*, de 1677, Baruch Spinoza (1980) dice algo así como que los seres humanos son tan soberbios de creer que el mundo fue hecho, fabricado y organizado para su satisfacción. No tener esto presente lleva a pensar que luego de portarnos mal con la madre naturaleza debemos pagar las consecuencias. Creo que esa economía de premios y castigos para entender el azote epidémico no es muy convincente. En otras palabras, las mutaciones genéticas no pueden explicarse única y exclusivamente de la mano de, digamos, la brutal tala de árboles – una práctica de muy larga data – o el calentamiento global – un problema, por decirlo de algún modo, más reciente cuya gravedad no deja de intensificarse. Si así fuera, no hay modo de explicar muchas de las epidemias del pasado. Al final de cuentas, las epidemias son tan viejas como la humanidad. Espero que se entienda lo que quiero decir: las enfermedades y epidemias son fenómenos sociales, culturales, biológicos, económicos, pero su origen es algo más complejo que no se explica solamente de la mano de la economía, la ecología o la cultura. Y esto es crucial en la historia de la ciencia y la medicina y, también, en las evaluaciones que se hacen de la performance de la salud pública y las políticas de salud.

La séptima lección: Sin ser la primera pandemia en tiempos de acelerada circulación de personas, productos, información e ideas, la covid-19 sí parece haber traído consigo una crisis planetaria y multidimensional, percibida en Occidente como única e incomparable. Se habla, entonces, de la pandemia de covid-19 como una crisis sin precedentes. Me pregunto si es esa la más convincente perspectiva histórica. ¿Será que cada generación vive algunos eventos extraordinarios de su presente – por ejemplo, una epidemia – como únicos, demarcatorios de un antes y un después? Ya indiqué que no creo que la historia ofrezca lecciones sobre cómo navegar el presente y el futuro. Solo perspectivas. Por ello me gustaría reafirmar que si hay algo que sí nos revela el pasado es que otras epidemias fueron tan o más trágicas que la que estamos viviendo. Esto, por supuesto, no debe servirnos como un consuelo. Pero sí puede servirnos para mirar lo que estamos viviendo con una sutil sensibilidad que, quiero creer, la historia debería poder ofrecernos.

Y una octava y última lección, referida a la cuestión de las vacunas. Hemos visto, y seguimos viendo, cuán complejos e intrincados son los asuntos asociados al descubrimiento de una vacuna y su uso como recurso de salud pública. Listo algunos, pertinentes al lugar periférico de América Latina en la producción de vacunas. Es innecesario decir que América Latina fue y es una periferia heterogénea, donde hubo y hay relevantes descubrimientos biomédicos, pero estos nunca tuvieron, comparativamente hablando, la relevancia de los producidos por la biomedicina en algunos de los países centrales. No digo esto para volver a traer el ya muy criticado difusionismo científico, con un centro dinámico y activo y una periferia pasiva. Me interesa destacar, en cambio, que lo interesante históricamente hablando son los ajustes y acomodaciones que marcan la llegada de una vacuna producida en el centro y su impacto en las específicas y variadas realidades nacionales latinoamericanas.

Entonces, cabe preguntarse:

¿Cómo se produjo la aceptación de una vacuna producida en Europa o los Estados Unidos en los círculos médicos y científicos locales?

¿Se trató de un producto de importación que en algún momento empezó a producirse localmente?

¿Qué nuevas demandas presupuestarias generó en las finanzas del Estado?

¿Asignar recursos para la vacuna significó postergar otras urgencias en materia de salud pública en general?

¿Cómo respondió la industria farmacéutica local?

¿Cómo pensó el Estado las vacunas: como un derecho, como una obligación, como una opción individual?

¿Cómo tomaron forma y se consensuaron esas opciones? ¿Se trató de posturas diversas resultantes de determinados ideales respecto de los derechos y libertades individuales? ¿O se trató de una cierta civilidad sanitaria – con el Estado en su centro – asentada en la aceptación de la vacuna como modo de lidiar con la salud individual y colectiva? ¿Esa aceptación es parte de una tradición nacional o regional? ¿Qué elementos jugaron un rol importante al momento de elegir una y no otra de estas perspectivas? ¿O se trató de perspectivas – la de los derechos individuales y la de la civilidad sanitaria colectiva – que no tienen por qué ser excluyentes?

¿Qué mecanismos de marketing social fueron utilizados a los fines de difundir la vacunación masiva? ¿Fueron las instituciones estatales de la salud pública las que ejecutaron las campañas de vacunación o, además de ellas, hubo otras instituciones – estatales, públicas o privadas – que también jugaron un decisivo rol?

¿Qué respuestas sociales generaron estas políticas de vacunación? ¿Hubo resistencias? Si las hubo, ¿de qué sectores de la sociedad, cuánto se las politizó, y con qué argumentos?

¿La distribución y acceso a la vacuna estuvieron imbricados por asuntos de corrupción, algo muy común en el presente, pero del que sabemos poco en el pasado?

* * *

Al empezar me referí a la sorpresa de Trump frente a la pandemia. También expresé mis reservas con los discursos de algunos filósofos, la audacia interpretativa de los periodistas que opinan de todo, y los desafíos de la política y los políticos frente al brutal azote de una epidemia.

Ahora cabe señalar que si la discreción, modestia y cautela pueden ser un bien escaso entre ellos, algunos historiadores han hecho todo lo posible para sumarse a ese grupo fuertemente tentado en vaticinar de la mano de la improvisación o explicar con generalizaciones y simplificaciones. Cierro estas notas con el comentario, para mi irritante, de uno de esos historiadores que creía tener una respuesta para todo lo que estamos viviendo. Decía:

> A más de un año de haberse declarado oficialmente que la enfermedad del covid-19 era una verdadera pandemia, … está cada día más claro que el causante principal de esta terrible pandemia, que azota actualmente a la humanidad entera, no es otra que el sistema mundial capitalista todavía vigente en escala planetaria. Ya que lejos de las

> tramposas pseudo-explicaciones naturalistas o biologicistas, que querían presentar al covid-19 como una azarosa e infortunada mutación 'natural' de un virus animal que migra, también de modo desafortunado y casual, hacia los seres humanos, se impone cada día más claramente la evidente realidad de que es más bien el capitalismo, con su profunda e ineludible lógica depredadora y destructora de la naturaleza y de la ecología, el que termina por provocar estas mutaciones y migraciones de las enfermedades animales hacia la cada vez más frágil y precaria especie humana (Aguirre Rojas, 2021).

No quiero, en modo alguno, que se lean estas notas como una entusiasta defensa del capitalismo. Pero si hay algo que los historiadores debemos tener es perspectiva. El siglo XIX, solo por poner un ejemplo, produjo grandes y muy imaginativas propuestas que ofrecían futuros deseados y grandes y muy generales síntesis de la experiencia humana en el pasado. Pero ocurre que después la historia hizo lo suyo. Y las cosas no fueron tal como se anticipaba o imaginaba, en gran medida porque la realidad es siempre más compleja que una formulación teórica o interpretativa. Fueron, en cambio, resultado de acciones humanas, colectivas e individuales y, también, a veces, resultado de factores extrahumanos, no siempre fácilmente controlables. Tal el caso de un virus. Esto último, me parece, es una dimensión clave de la que no podemos olvidarnos cuando intentamos entender qué pasó con las epidemias y las enfermedades en el pasado.

## NOTA

* Estas notas fueron leídas en ocasión de la celebración de los 20 años del Seminario de Historia de la Salud y la Enfermedad del Instituto de Investigaciones Históricas de la Universidad Nacional Autónoma de México, realizado el 12 y 13 de mayo de 2022. Retoman cuestiones planteadas en una serie de textos escritos en tiempos de pandemia (Armus, 2022, jul.-dic. 2022, 2020).
